# Community involvement in design, implementation and evaluation of nutrition interventions to reduce chronic diseases in indigenous populations in the U.S.: a systematic review

**DOI:** 10.1186/s12939-018-0829-6

**Published:** 2018-08-13

**Authors:** Jinan Banna, Andrea Bersamin

**Affiliations:** 1 Department of Human Nutrition, Food and Animal Sciences at the University of Hawai’i at Mānoa, Honolulu, USA; 2 Center for Alaska Native Health Research, Institute of Arctic Biology at the University of Alaska Fairbanks, Fairbanks, USA

## Abstract

**Background:**

Indigenous peoples of the United States disproportionately experience chronic diseases associated with poor nutrition, including obesity and diabetes. While chronic disease related health disparities among Indigenous people are well documented, it is unknown whether interventions adequately address these health disparities. In addition, it is unknown whether and to what extent interventions are culturally adapted or tailored to the unique culture, worldview and nutrition environments of Indigenous people. The aim of this review was to identify and characterize nutrition interventions conducted with Indigenous populations in the US, and to determine whether and to what degree communities are involved in intervention design, implementation and evaluation.

**Methods:**

Peer-reviewed articles were identified using MEDLINE. Articles included were published in English in a refereed journal between 2000 and 2015, reported on a diet-related intervention in Indigenous populations in the US, and reported outcome data. Data extracted were program objectives and activities, target population, geographic region, formative research to inform design and evaluation, partnership, capacity building, involvement of the local food system, and outcomes. Narrative synthesis of intervention characteristics and the degree and type of community involvement was performed.

**Results:**

Of 1060 records identified, 49 studies were included. Overall, interventions were successful in producing changes in knowledge, behavior or health (79%). Interventions mostly targeted adults in the Western region and used a pre-test, post-test design. Involvement of communities in intervention design, implementation, and evaluation varied from not at all to involvement at all stages. Of programs reporting significant changes in outcomes, more than half used at least three strategies to engage communities. However, formative research to inform the evaluation was not performed to a great degree, and fewer than half of the programs identified described involvement of the local food system.

**Conclusions:**

The extent of use of strategies to promote community engagement in programs reporting significant outcomes is notable. In planning interventions in Indigenous groups, researchers should consider ways to involve the community in intervention design, execution and evaluation. There is a particular need for studies focused on Indigenous youth in diverse regions of the US to further address diet-related chronic conditions.

## Background

Indigenous peoples of the United States disproportionately experience chronic diseases associated with poor nutrition, including obesity and diabetes. American Indian and Alaska Native (AIAN) preschool children have the highest prevalence of obesity compared to other racial/ ethnic groups (37.0% among AIAN compared to 17.4% among non-Hispanic whites) [1]. Disparities persist into adulthood, and recent reports indicate that the prevalence of obesity among AIAN adults is approximately 80% higher than among non-Hispanic whites and Asians [2]. Furthermore, although the prevalence of obesity appears to have leveled off in other ethnic and racial groups, the prevalence continues to rise among AIAN people [3, 4]. Consistent with obesity rate, the prevalence of diabetes is considerably higher in AIAN (17.9%) than in non-Hispanic whites (7.9%) [5].

While chronic disease related health disparities among AIAN people are well documented, it is unknown whether interventions adequately address these health disparities. In addition, it is unknown whether and to what extent interventions are culturally adapted or tailored to the unique culture, worldview and nutrition environments of AIAN people. Genuine and equitable partnerships between researchers and AIAN communities are critical to ensuring that interventions are relevant. Community based participatory research (CBPR) is considered best practice for conducting research with AIAN communities [6, 7]. Using a CBPR approach that draws on the traditional knowledge of communities can engender strength-based interventions that reinforce cultural continuity and have broad positive impacts. For example, programs seeking to strengthen the traditional food system have the potential to address high prevalence of chronic disease in addition to promoting food sovereignty. These traditional practices also provide a foundation for cultural identity, a basis for social support networks, and assist Indigenous Peoples in gaining greater autonomy [8].

While involvement of the community at every step of the process in intervention planning and implementation in Indigenous groups has been widely noted to be important in promoting adequate dietary intake, not all programs have involved engagement with community members. Examining and characterizing interventions that have involved community engagement to various degrees may prove useful for researchers and educators planning to implement programs in Indigenous communities. The primary objectives of this systematic review were to: 1) Identify and characterize nutrition interventions conducted with Indigenous populations in the United States; and 2) To determine whether and to what degree communities are involved in the design, implementation, and evaluation of the intervention.

## Methods

Peer-reviewed journal articles were identified using the online database MEDLINE.

### Study inclusion and exclusion criteria

To be included in the review, research articles had to 1) be published in a refereed journal between 2000 and 2015; 2) report on a diet-related program or intervention conducted in an Indigenous population in the United States; 3) report outcome data. Studies that were reported in a language other than English, reported on process data only, and review papers were not included.

### Search strategy

The following two overarching concepts were identified to guide selection of search terms and phrases based on the review objectives: Indigenous populations and chronic disease prevention/health promotion. For the first key concept, related terms and phrases identified were: ‘*Hawaii/ethnology*,’ ‘*Native Hawaiian*,’ ‘*Indians, North American*,’ ‘*American Indian*,’ ‘*Native American*,’ ‘*Native Alaskan*,’ ‘*Oceanic Ancestry Group/ethnology*.’ For the second key concept, search terms and phrases were as follows: ‘*diabetes mellitus/education*,’ ‘*diabetes mellitus/prevention and control*,’ ‘*chronic disease/prevention and control*,’ ‘*nutritional sciences/education*,’ ‘*obesity/prevention and control*,’ ‘*health promotion*,’ ‘*overweight/prevention and control*,’ ‘*cardiovascular diseases/prevention and control*,’ ‘*wellness*,’ and ‘*intervention*.’ Boolean operators such as AND and OR were used to link search terms.

**Fig. 1:**
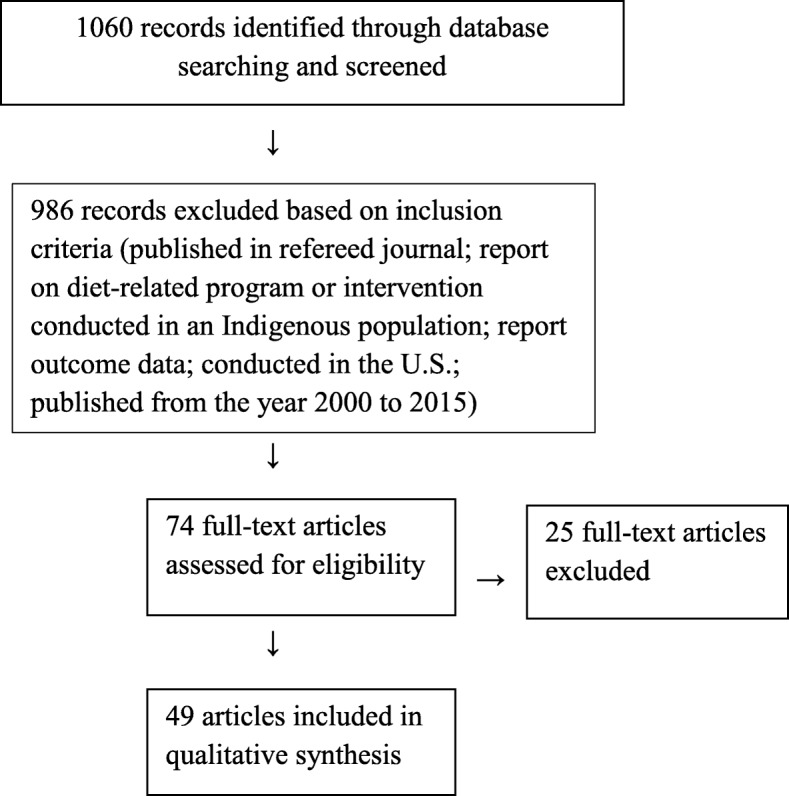
Flow Diagram Illustrating Selection of Studies for Systematic Review of Community Involvement in Design, Implementation and Evaluation of Nutrition Interventions in Indigenous Populations in the U.S

Using the search terms, one researcher (JB) identified abstracts for review. Referencing the inclusion criteria, the same researcher examined the abstracts and excluded studies not meeting the requirements for inclusion. The same procedure was followed with the full papers identified using the abstracts remaining. Finally, for all full papers included, a search was conducted for other papers reporting on the same study by searching for the title of the intervention and examining the references in each paper. This search was conducted to address the fact that many papers reporting outcomes do not report on the steps involving community engagement. While one goal of the review was to characterize interventions and thus gauge the effectiveness of programs through outcomes reported, it was also imperative to capture community involvement through examination of all papers related to each study. Figure 1 illustrates the search strategy used for this review.

### Data extraction

After obtaining consensus on the abstraction tool, two researchers (JB, AB) piloted the tool on two studies to reach agreement on the procedures for data abstraction. One researcher (JB) then conducted a systematic review of full studies identified and completed the abstraction table. Data entered in the abstraction table included the following: study objectives, target population/inclusion criteria, study design, study setting (city, state), study duration, sample size, response rate, intervention (focus and main activities of the program), outcome variables, measures utilized, involvement of the community in the design, implementation, and evaluation of the intervention (formative research to inform design and evaluation, partnership, community capacity building, involvement of local food system), treatment length/follow up, drop out, results, main implications, and limitations.

### Data synthesis

Table 1 contains a list and description of the 49 studies identified in the literature search. Within these 49 studies, there were 39 distinct programs, given that the outcomes of some programs were described in multiple publications.

**Table 1:** List and description of the 49 studies identified in the literature search

Program Title	Author(s)	Study Design	Target Population, Participant No. and Characteristics and Age Group	Objective	Activities	Outcomes and Impact				
						Diet/Food Behaviors	PA	Weight Status	Knowledge/Awareness/ Self-efficacy	Other Health Outcome
Bright Start study	Story et al. [9]^a, b^	Group-randomized school-based trial	Kindergarten and first grade Lakota American Indian Children in 14 schools on Pine Ridge reservation in South Dakota (*n* = 454)	To develop and test the effectiveness of a school environment intervention, supplemented with family involvement, to reduce excessive weight gain by increasing physical activity and healthy eating practices among kindergarten and first-grade American Indian children.	Physical activity intervention: school PE, class walks outdoors, in-class action breaks, and active recess. Dietary intervention: foodservice staff trained to offer 1% white milk instead of 2% or whole milk, eliminate chocolate or other flavored milks, serve recommended portion sizes, purchase and use low-calorie/fat foods, provide more fruits, etc. Behavioral messages delivered at family events: eat more fruits and vegetables, substitute water for sugar-sweetened beverages, limit high-fat and high-sugar snacks and fast foods, drink skim or 1% milk, promote physical activity, and limit TV and video time.	✓^c^	✓^d^	✓^c^		
	Arcan et al. [36]^b^	Pre-post study (sub-study)	Kindergarten and first grade teachers in 14 schools in South Dakota (*n* = 75)	To examine teachers’ classroom and school food practices and beliefs and the effect of teacher training on these practices and beliefs	Interactive training to explain causes and implications of childhood obesity	✓^c^				
Cancer 101	Hill et al. [17]^a^	Longitudinal, pre-post study	Adults who were members of 43 tribes in the Pacific northwest, health professionals and community health representatives working with NA populations (*n* = 70)	To examine the impact of Cancer 101, a cancer education resource developed in collaboration with American Indians/Alaska Natives to improve cancer knowledge, action regarding cancer control in tribal settings, and survival rates for members of their communities	Topics addressed: [1] overview of the cancer concern among the AI/ AN population, [2] what is cancer?, [3] cancer screening and early detection, [4] cancer diagnosis and staging, [5] cancer risks and risk reduction, [6] basics of cancer treatment, [7] support for patients and caregivers.				✓^c^	
Cherokee Choices	Bachar et al. [37]^a^	Pre-post study	Eastern Band of Cherokee Indians (EBCI) children (n = 55 for mentoring and after-school program; *n* = 85 for mentoring only) and adults (*n* = 86) in a worksite wellness program in 1 community in North Carolina	To develop and evaluate a community- based intervention to improve the health of a rural, mountainous community in North Carolina	Elementary school mentoring, worksite wellness for adults, and church-based health promotion. A social marketing strategy, including television advertisements and a television documentary series, supports the three components.	✓^e^	✓^e^	✓^e^		
Community Health Promotion Grants Program (CHPGP)	Wagner et al. [38]^b^	Pre-post study	11 communities, including three rural/suburban counties, two Native American regions or reservations, four cities, and two states in the western United States	To present results from an outcome evaluation of the CHPGP in the West, which represented a major community-based initiative designed to promote improved health by changing community norms, environmental conditions, and individual behavior in 11 western communities.	A media campaign, nutrition education campaign in grocery stores, and school-based nutrition education	✓^d^			✓^c^	
Community Health Worker intervention	Beckham et al. [39]^a^	Pre-post study	Native Hawaiian and Samoans on Oahu, 18 years of age or older, diagnosis of type 2 diabetes and HbA1c readings over 10.0%; *n* = 116: *n* = 80 CHW intervention, *n* = 36 No CHW intervention	To examine the effectiveness of CHWs on diabetes management among a population with primarily Native Hawaiian and Samoan ethnic minority participants with HbA1c greater than 10%.	CHWs conducted diabetes self-management education for participants. Participants were given voluntary access to the Traditional Hawaiian Healing Center.					✓^c^
Diabetes Prevention Program (DPP)	Mayer-Davis et al. [40]	RCT	Diverse adults age > 25 years, BMI > 24 kg/m2 (> 22 kg/m2 for Asian Americans), at high risk for diabetes (specifically, impaired glucose tolerance plus a fasting plasma glucose of 5.3–6.9 mmol/l [or ≤ 6.9 mmol/l for American Indians]). recruited from 27 clinical centers throughout the US (*n* = 2934)	To describe usual dietary intake assessment at baseline and 1-year post-randomization in the ethnically diverse DPP cohort.	Training in diet, exercise and behavior modification skills, frequent contact with the interventionist, and delivery of the intervention in a culturally sensitive manner.	✓^c^				
	Wing et al. [41]	RCT (secondary analysis)	Participants assigned to the intensive lifestyle intervention who were at least 25 years, BMI of 24 kg/m2 or greater (22 kg/m2 or higher in Asians), a fasting plasma glucose of 95 to 125 mg/dL (125 mg/dL in Native Americans) and a plasma glucose value of 140 to 199 mg/dL 2 h after a 75-g glucose load from 27 clinical centers throughout the US (*n* = 1079).	To examine demographic, psychosocial, and behavioral factors related to achieving weight loss and physical activity goals in the DPP lifestyle participants.	Participants were assigned an individual case manager. Core curriculum introduced basic skills related to nutrition, exercise, and behavior change.		✓^e^			
Family Education Diabetes Series (FEDS)	Mendenhall et al. [42]^a^	Pre-post study	American Indian adults in a Midwestern community with type 2 diabetes (n = 36).	To describe the FEDS as an example of CBPR, and highlight pilot findings assessing its value and impact across key diabetes-relevant variables.	Members check and record each other’s blood sugars, weight, and conduct foot checks. Participants cook and eat meals together consistent with AI cultures and traditions and discussion follows regarding the meal’s ingredients, cost and availability, portion sizes, and relevance to diabetes and healthy weight maintenance. Educational sequences follow in talking circles on basic diabetes education, obesity and weight loss, foot care, stress management, exercise, etc.			✓^c^		✓^c^
Five a Day, the Rio Grande Way	Buller et al. [43]^a^	Pre-post study embedded in RCT	Rural Multicultural adults in the Upper Rio Grande Valley living at least 6 months or more in the region, at least 18 years of age, and able to speak and read English; *n* = 755 (380 in intervention, 373 in control group). Two-thirds were Hispanic, 9% AIAN	To evaluate effectiveness of a website promoting fruits and vegetables in the rural multicultural Upper Rio Grande Valley.	The 5 a Day, the Rio Grande Way website was created for this study to encourage fruit and vegetable consumption in the region. Participants were instructed to log on to the website at least once a month over the 4-month study period.	✓^c^				
Hawai‘i Community Resource Obesity Project: Lifestyle Enhancement Program	Bradley et al. [44]^a^	Quasi experimental pre-post study	Over half of participants (58%) were of Native Hawaiian descent; 18 years of age or older, BMI > 30 kg/m2; Intervention group: *n* = 217 (158 women, 59 men); Control group: *n* = 89	To describe the effects of a community-based, multidisciplinary team-led, lifestyle modification program on short-term weight loss in a morbidly obese predominantly Native Hawaiian population.	Physical activity, nutrition education, and behavioral counseling.			✓^c^		
Healthy Foods Hawaii	Gittelsohn et al. [45]^a, b^	Pre-post study	Children and their adult caregivers in two low-income multiethnic communities in Hawaii (*n* = 174 caregiver-child pairs)	To decrease risk for obesity in children by modifying the food environment and conducting point-of-purchase promotions that will lead to changes in psychosocial factors and behaviors associated with healthier food choices among low-income communities with a preponderance of Native Hawaiians and Pacific Islanders	The intervention aimed to increase the availability of healthy foods in stores in the two intervention communities, and promote healthier food choices and food preparation methods. The intervention comprised of four phases (healthier beverages, healthier snacks for children, healthier condiments, and healthier meals).	✓^c^			✓^c^	
Healthy Living in Two Worlds	Weaver et al. [12]^a^	Pre-post study	Young urban Northeastern population, the Haudenosaunee (six interrelated Native American nations) people (*n* = 16).	To report the results of the initial delivery of the Healthy Living in Two Worlds curriculum, which sought to increase physical activity, decrease or prevent recreational tobacco use, and increase healthy eating practices.	The curriculum was implemented using a summer day camp format and included field trips and guest presenters covering topics such as Haude-nosaunee dance skills, lacrosse skills, and the traditional role of tobacco in Native American societies.	✓^d^				
Ho-Chunk Youth Fitness Program	Carrel et al. [46]	Pre-post study	Children aged 6–18 years, native and non-native to the Ho-Chunk Tribe in Wisconsin; *n* = 38	To evaluate whether risk of type 2 diabetes can be decreased by interventions that affect the lifestyles of children at high risk.	Classes with supervision for both nutrition and exercise, including fitness and nutrition education, physical activity, and making a healthy snack.			✓^d^		✓^c^
Journey to Native Youth Health Program	Brown et al. [47]^a^	RCT	Northern Plains Indian youth 10–14 years old in Montana (*n* = 76)	To develop a lifestyle change program for Native American youth by modifying the Diabetes Prevention Program (DPP) and assess implementation indicators and short term behavioral and physiological outcomes of the intervention among a small pilot sample.	The Journey DPP included 9 small group sessions led by a Native American community member. Cultural activities, hands on activities, keeping and discussing weekly activity and nutrition diaries, and group games were incorporated throughout the program.	✓^d^	✓^d^	✓^d^		
Ke’ Ano Ola (KAO)	Gellert et al. [48]	Pre-post study	Individuals representing the ethnic and socioeconomic status of the Molokai community: 64% Native Hawaiian, 25% Filipino, 5% white (*n* = 74).	To evaluate a community-based 12-week healthy lifestyle program in Molokai which was developed to decrease chronic disease risk through health education emphasizing weight loss, exercise and risk factor reduction.	KAO participants attended sessions. Each week the program leader introduced an educator who would conduct an interactive educational session focusing on 1 aspect of chronic disease prevention. The second class focused on physical activity.			✓^c^		✓^c^
Life in BALANCE pilot study	Benyshek et al. [49]	Pre-post study	Urban American Indian/Alaskan Native (AI/ANs) residents in Las Vegas, Nevada. 21 years of age or older, AI/AN self-identification, BMI ≥ 25 kg/m2, HbA1c level between 5.4 and 6.4% (*n* = 22)	To describe the translational type 2 diabetes prevention program specifically modified for an urban AI/AN population and utilize the results of the pilot project to inform larger future type 2 diabetes prevention programs targeting urban AI/AN populations through diet modification and increased physical activity.	Type 2 diabetes prevention core curriculum and regular follow up. Curriculum included: weight-loss curriculum, meal planning, fat gram and calorie counting, portion size, and food content education.			✓^c^		✓^c^
Medicine Wheel Nutrition Intervention	Kattelmann et al. [50]^a^	RCT	Northern Plains Indians from Cheyenne River Sioux Tribe in South Dakota aged 18 to 65 years, with type 2 diabetes (*n* = 114).	To determine if Northern Plains Indians with type 2 diabetes mellitus who are randomized to receive culturally adapted educational lessons based on the Medicine Wheel Model for Nutrition in addition to their usual dietary education will have better control of their type 2 diabetes than a nonintervention, usual care group who received usual dietary education from personal providers.	The education group received nutrition lessons based on the Medicine Wheel Nutrition Model to encourage consumption of a diet patterned after the traditional consumption of macronutrients for Northern Plains Indians; protein (25% of energy), moderate in carbohydrate (45 to 50% of energy), low in fat (25 to 30% of energy). A registered dietitian and tribal member, trained in the curriculum, led the classes.	✓^d^	✓^d^	✓^c^		
Native American Cardiovascular Risk Reduction Program	Burden et al. [51]	Retrospective study	Native Americans/ All patients enrolled in the cardiovascular risk reduction program from March 1997–October 1999. Populations of 11 Rio Grande pueblos and numerous Native Americans living in urban areas in New Mexico; n = 74 (36 men, 38 women)	To report lipid management outcomes associated with a cardiovascular risk reduction program for Native Americans	Each patient enrolled in the program were managed by a pharmacist clinician (PhC)-registered dietitian team. The primary nutrition intervention strategy utilized the American Heart Association diet in conjunction with increases in dietary fiber and physical activity.					✓^c^
Native Hawaiian Diabetes Intervention Program	Mau et al. [52]	Quasi-experimental	NHs age 30 years with diabetes or high risk for diabetes in two rural communities in Hawai’i (n 147)	To examine the association of stage of change with diet and exercise behaviors in response to a lifestyle intervention for NHs.	Participants received the ‘ohana support (OS) (in which a support person attended all activities with them) or the standard intervention from community peer educators.	✓^d^	✓^d^			
Navajo Healthy Stores program	Gittelsohn et al. [53]^a, b^	RCT	Adult Navajo consumers (baseline, *n* = 276; postintervention, *n* = 145)	To examine the impact of the NHS program on Navajo community members by measuring changes in psychosocial variables, food-related behavior, and BMI.	The intervention sought to increase availability of healthier foods in local food stores and to promote these foods at the point of purchase and through community media.			✓^c^	✓^d^	
Partners in Care	Sinclair et al. [54]^a^	Pre-post study embedded in RCT	Self-reported physician-diagnosed type 2 diabetes, 18 years of age or older, self-reported Native Hawaiian, Filipino, or other Pacific Islander, English-speaking, and baseline A1c > 7% in Hawaii (*n* = 82)	To pilot test the effectiveness of a culturally-adapted diabetes self-management intervention.	Curriculum materials emphasized American Diabetes Association clinical guideline goals for blood glucose, A1c, blood pressure, and lipids. Group meetings were delivered by trained peer educators				✓^c^	✓^c^
Pathways	Caballero et al. [55, 56]^a, b^	RCT	American Indian schoolchildren followed from 3rd to 5th grade; *n* = 1704 children in 41 schools in Arizona, New Mexico, and South Dakota	To evaluate the effectiveness of a school-based, multicomponent intervention for reducing percentage body fat in American Indian schoolchildren.	The intervention had 4 components: 1) change in dietary intake, 2) increase in physical activity, 3) a classroom curriculum focused on healthy eating and lifestyle, and 4) a family involvement program.	✓^c^	✓^d^	✓^d^	✓^c^	
	Cunningham-Sabo et al. [57]^a, b^	RCT	American Indian schoolchildren at 39 schools	To reduce fat content of school meals to 30% or fewer calories from fat without compromising dietary quality.	The food service intervention included the development of nutrient guidelines operationalized as behavioral guidelines. These behavioral guidelines included specific steps and skill-building techniques for lowering the fat content of menu items. To support the behavioral guidelines, training sessions were conducted with all food service staff. These training sessions were reinforced by kitchen visits in the first year and additional visits to each school in the second and third years.	✓^c^				
	Davis et al. [58]^a, b^	RCT	American Indian schoolchildren (*n* = 1704) in 3rd to 5th grade from 41 schools in New Mexico, Arizona, Utah, and South Dakota	To promote healthful eating and increased physical activity in seven American Indian communities.	The classroom curricula incorporated culturally appropriate lessons through the use of tribal knowledge, maps of Pathways Nations, and AI stories comprising in-class and take-home activities that promoted healthful eating behaviors and increased physical activity. The family component of the intervention included two main family intervention strategies: 1) Information reinforcing a health education message taught in the classroom curricular lessons and healthful nutrition tips; 2) Events with interactive booths, printed educational materials, and displays that included educational and behavioral messages about low-fat foods and physical activity.			✓^d^	✓^c^	
	Himes et al. [59]^b^	Pre/Post for school lunch observations. Post-test only for 24-h recalls.	American Indian school grade [grade 2 (baseline) to grade 5 (follow-up)] children representing 7 American Indian Nations; Direct Observation: *n* = 470. 24-h recalls at follow-up for the whole day: *n* = 620.	To report the impact of the Pathways intervention on diet, using data from two sources: the direct observation of children eating school lunch, and 24-h dietary recalls.	The intervention included a school curriculum, a physical activity/physical education component, a school foodservice component, and a family component. The nutritional aspects of the curriculum and family component emphasized healthy eating and low-fat food alternatives. The food- service intervention focused on nutrient and behavioral guidelines to reduce the amount of fat in school meals.	✓^c^				
	Stevens et al. [60, 61]^a, b^	RCT	American Indian school children from 41 schools located in Arizona, New Mexico, and South Dakota (*n* = 1447)	To examine the impact of the Pathways intervention on psychosocial variables related to physical activity and diet in American Indian children.	Four components: (a) food service intervention which modified foods served in the school cafeteria; (b) physical education component which increased physical activity at school; (c) classroom curriculum that focused on knowledge and practices related to healthy eating and lifestyle habits; and (d) family component aimed at involving parents of children participating in the program, to create a positive and supportive environment for modifying dietary practices and physical activity.		✓^c^		✓^c^	
	Stone et al. [62]^a, b^	RCT	American Indian third grade students in seven different American Indian communities in Arizona, New Mexico, and South Dakota (*n* = 1704)	To provide a brief overview of the Pathways study design and results.	Four components: (a) food service intervention which modified foods served in the school cafeteria; (b) physical education component which increased physical activity at school; (c) classroom curriculum that focused on knowledge and practices related to healthy eating and lifestyle habits; and (d) family component aimed at involving parents of children participating in the program, to create a positive and supportive environment for modifying dietary practices and physical activity.	✓^c^	✓^c^	✓^d^	✓^c^	
	Story et al. [63]^a, b^	RCT	American Indian third grade students in seven different American Indian communities in 41 schools in South Dakota (n = 1704)	To evaluate the Pathways food service intervention, designed to lower the fat in school lunches over a 3-year period.	Food service intervention which modified foods served in the school cafeteria in line with the Dietary Guidelines for Americans.	✓^c^				
Phoenix Indian Medical Center (PIMC) Breastfeeding Support Program	Murphy et al. [13]^a^	Pre-post study	NA new mothers in the southwestern United States (*n* = 5556)	To evaluate an innovative program that targets promotion of breastfeeding among Native women as a type 2 diabetes prevention intervention.	A registry of new mothers delivering or entering care at the medical center was maintained to ensure that services were provided to as many mothers as possible, track feeding choices, and ensure follow-up. The PIMC also implemented a toll-free phone service for patients and staff to call for information and support. Regularly scheduled breastfeeding education classes for women and their families and for health care professional staff were initiated.	✓^c^				
PILI ‘Ohana Pilot Project	Mau et al. [14]^a^	Pre-post study	NH, Filipino or OPI overweight/ obese adults ≥18 years or older in Hawai’i (*n* = 239)	To implement and examine the effectiveness of the culturally-adapted Diabetes Prevention Program Lifestyle Intervention (DPP-LI) to promote weight loss in 5 NHOPI communities.	Community peer educators delivered lessons on lifestyle change, being active, healthy eating, managing stress, and communicating with health professionals.			✓^c^		
	Kaholokula et al. [64, 65]^a^	Pre-post study	Native Hawaiian, Filipino or other Pacific Islander ancestry (i.e., Chuukese, Samoan), 18 years of age, overweight/obese in Hawaii (*n* = 239).	To elucidate the sociodemographic, behavioral, and biological factors associated with early weight-loss efforts among four ethnically and organizationally diverse groups with members at high risk for diabetes.	The POP translated the Diabetes Prevention Program Lifestyle Intervention (DPP-LI) to initiate weight- loss efforts as part of a larger community-based lifestyle intervention. The intervention focused on evidence- based behavioral weight loss strategies for healthy eating, physical activity and stress/negative emotions management utilizing individual action planning.	✓^c^	✓^c^	✓^c^		✓^c^
Sacred Beginnings	Richards et al. [66]^a^	Pre-post study embedded in RCT	AI girls aged 11–14 years old in the Northern Plains region (*n* = 77)	To examine the influence of a CBPR preconception health intervention among AI adolescent females	The educational intervention consisted of 15 preconception health education sessions and was piloted during a summer high school residential academic program. Topics included nutrition; fitness and exercise; diabetes; prescription medication abuse; Lakota cultural perspectives on womanhood, pregnancy, and parenting.				✓^c^	
Special Diabetes Program for Indians Diabetes Prevention (SDPI-DP)	Jiang et al. [67]^a^	Pre-post study	AI and AN at least 18 years of age, no previous diagnosis of diabetes, and having either impaired fasting glucose (IFG) or impaired glucose tolerance (IGT) (*n* = 2553).	To evaluate a translational implementation of the Diabetes Prevention Program (DPP) (showing that lifestyle intervention can prevent or delay the onset of diabetes for those at risk) intervention in a diverse set of American Indian and Alaska Native (AI/ AN) communities.	The curriculum was delivered in group settings. It was supplemented by individual lifestyle coaching sessions to individualize goals and plan and to identify and solve barriers to participation. Participants were encouraged to use a Keeping Track booklet to monitor their fat and calorie intake and weekly physical activity. If used, booklets were reviewed by lifestyle coaches who gave feedback to the participants during the lifestyle coaching sessions.			✓^c^		✓^c^
Special Diabetes Program for Indians Healthy Heart Demonstration Project	Moore et al. [68]	Pre-post study	AI/AN 18 years or older with diabetes (*n* = 3373)	To evaluated the effect on multiple CVD disease risk factors among AI/ANs with diabetes who participated in a large-scale, multidisciplinary, intensive case management intervention.	Multidisciplinary teams implemented an intensive case management intervention. The case manager developed an individualized care plan for CVD risk reduction for each participant and periodically updated it in response to participant progress.					✓^c^
Stanford Chronic Disease Self-Management Program (CDSMP)	Jernigan et al. [69]^a^	Post-test	Native American adults, aged 18 years and older, with Type 2 diabetes in the Santa Clara Valley (*n* = 12)	To demonstrate how the CBPR process facilitates the successful translation of the Stanford program and how CBPR is used within this community to build community capacity.	The Stanford CDSMP is a peer-led program that integrates self-efficacy theory through guided mastery experiences, acquisition of skills and enhancement of self-confidence through peer modeling, reinterpretation of physiological symptoms, and social persuasion.		✓^e^			
Stop Atherosclerosis in Native Diabetics Study (SANDS)	Howard et al. [70, 71]	Pre-post study	AI men and women (*n* = 548) with type 2 diabetes, aged 40 years or older in Oklahoma, Arizona, and South Dakota	To compare progression of subclinical atherosclerosis in adults with type 2 diabetes treated to reach aggressive targets of low-density lipoprotein cholesterol (LDL-C) of 70 mg/dL or lower and systolic blood pressure (SBP) of 115 mmHg or lower vs standard targets of LDL-C of 100 mg/dL or lower and SBP of 130 mmHg or lower.	Study personnel performed BP and lipid management. Participants receive nutrition counseling and physical activity recommendations consistent with Indian Health Service (IHS) guidelines for people with diabetes, hypertension, and dyslipidemia, and were referred to IHS provider for diabetes management.					✓^c^
Strong in Body and Spirit	Gilliland et al. [11]^a^	Nonrandomized community-based intervention	Native Americans ≥ 18 years with type 2 diabetes in New Mexico (*n* = 159)	To determine the effects of a culturally appropriate diabetes lifestyle intervention for Native Americans with risk factors or complications of diabetes.	The intervention was designed to improve participants’ overall risk factor profiles and focused on making healthy changes (e.g. eating less sugar and fat, more exercise) and was not a weight -loss program.					✓^c^
Toddler overweight and tooth decay prevention study (TOTS)	Karanja et al. [72]^b^	Pre-post study	Expectant mothers and their families recruited from three AI tribes in Portland assigned to a community-wide intervention alone (tribe A; *n* = 63 families) or community-wide intervention containing a family component (tribes B and C; *n* = 142 families).	To assess the feasibility, acceptability and efficacy of these approaches in preventing toddler overweight.	Community-wide interventions used five strategies: (a) raising awareness (b) providing health education (c) facilitating individual behavior change, (d) augmenting public health practice and (e) modifying environments and/or policies related to breastfeeding, sugar-sweetened beverages and water consumption.			✓^c^		
Uli’eo Koa Program	Leslie et al. [73]	Pre-post study	12 Native Hawaiian adult males and 4 adult females ages 22–64 years old in Hawaii. All were active members of a Hawaiian group that practiced a traditional Hawaiian art of fighting	To demonstrate the efficacy of the program in maintaining and increasing physical fitness levels of moderately active Native Hawaiian adults.	Phase I consisted of daily exercise and meals- provided 3 daily meals and snacks. Phase II reduced meals to 2 evening meals per week. PA was less frequent and participants were responsible for the level of exercise they desired to maintain and responsible on their own for all other meals.	✓^e^				
Well-Integrated Screening and Evaluation for Women Across the Nation (WISEWOMAN)	Witmer et al. [74]	Pre-post study embedded in RCT	AN and AI women aged 40–64 who lived on the road system within a 50-mile radius of Anchorage (*n* = 76)	To describe the design and implementation of a heart disease prevention pilot program tailored for Alaska Native women, pilot study outcomes, and modifications made to the study protocol to increase the feasibility of large-scale implementation.	The intervention included weekly sessions on lifestyle change and goal setting		✓^c^	✓^d^		✓^d^
Zuni high school diabetes prevention progra	Ritenbaugh et al. [10]^a, b^	Multiple cross-sectional study	Zuni (*n* = 72) and Anglo (*n* = 37) high school youth of Zuni Pueblo in New Mexico	To test the feasibility and efficacy of a high school-based diabetes prevention intervention.	The prevention program included an educational component targeting decreased consumption of sugared beverages, knowledge of diabetes risk factors, and a youth-oriented fitness center			✓^d^		✓^c^
N/A	DeWeese et al. [75]^a^	Pre-post study	Tribal leaders, fish harvesters, women of childbearing age, children, and elders, as well as the broader tribal population in the Great Lakes Region; *n* = 193 (fish consumption study) (23 tribal members 15 males and 8 females from 5 of 6 GLIFWC (The Great Lakes Indian Fish and Wildlife Commission) member tribes for focus groups for the Advisory Map Development intervention)	To determine whether a map based advisory program (GLIFWC) would affect harvest and consumption behaviors in ways to reduce exposure to methyl mercury in Ogaa (Walleye) to levels that do not exceed the EPA RfD, while maintaining an important Anishinaabe tribal lifeway (the harvest and consumption of Ogaa).	Dissemination of advisory maps to target groups via oral presentations which included detailed training on use of maps, general information about adverse health effects of mercury exposure, information on how map-based consumption advice was developed.				✓^c^	
N/A	Driscoll et al. [76]	Pre-post study	Residents in 3 communities of Robeson County, North Carolina. African American Residents of the township of Redsprings, Hispanic residents of St. Pauls, and Native American (Lumbee Tribe) residents of Lumberton, North Carolina (*n* = 194). All participants were aged 18 years or older, fluent in either English or Spanish, and had caught or consumed local fish at least once in the previous year.	To assess the determinants of subsistence fishing and promote informed fish consumption among culturally distinct and lower income subsistence fishers in south eastern North Carolina.	Three sets of fish advisories, in the form of trifold brochures, were developed base on formative data collected in each community. Brochures provided info necessary to make informed fish consumption decisions. Brochures were evaluated to assess immediate pre/post changes.				✓^e^	
N/A	Harvey-Berino et al. [15]	Pre-post study	Native American mothers in New York, Quebec, and Ontario with BMI over 25 kg/m [2] and children between 9 months and 3 years.	To determine whether maternal participation in an obesity prevention plus parenting support (OPPS) intervention would reduce the prevalence of obesity in high-risk Native-American children when compared with a parenting support (PS)-only intervention.	Subjects were randomly assigned to one of two treatment groups: PS or OPPS. Subjects in both conditions participated in a program conducted by an Indigenous peer educator in the home of each participant.			✓^d^		
N/A	Rinderknecht et al. [16]^a^	Single-group pretest, posttest	104 urban Native American youth (65 children and 39 adolescents) attending an after-school program in Minneapolis.	To improve dietary self-efficacy through a 7-month nutrition intervention for Native American children (5 to 10 years) and adolescents (1 l to 18 years).	Lessons focused on improving self-efficacy by exposing the youth to more healthful foods, providing opportunities for the youth to successfully choose the more healthful alternatives (taste-testing), discussing ways to achieve balance through healthful eating and physical activity, and conducting the participative learning activities with peer groups, allowing modeling opportunities.				✓^c^	
N/A	Smith et al. [77]	Pre-post study	WIC clients from the Aleutian Islands (51% Alaskan Aleut, 27% white Non-Hispanic, 15% Asian, and 6% Hispanic) (*n* = 400). Other ethnic groups represented were Filipino, Eskimo, and Native American. Educational back- ground varied from high school or less to graduate degrees; English was a second language for most.	To get a better understanding of how television can serve a Native population to enhance nutrition education.	Fifteen 30-min programs were produced featuring a central nutrition theme consistent with WIC project objectives and were broadcasted throughout the summer through state- supported Alaska Rural Communication Systems.				✓^c^	
N/A	Thompson et al. [78]	Pre-post study embedded in RCT	Young urban American Indian women aged 18–40 years in New Mexico (*n* = 200)	To describe the effects of a culturally influenced intervention on behavioral risk factors for type 2 diabetes among asymptomatic AI women recruited from the general urban community.	Five discussion group sessions were held focusing on healthful eating, physical activity, goal-setting, and social support.	✓^c^				

*AI* American Indian, *AN* Alaska Natives, *RCT* randomized controlled trial, *CHW* community health worker, *CBPR* community-based participatory research, *NA* Native American, *NH* Native Hawaiian, *NHOPI* Native Hawaiian and Other Pacific Islander, *BMI* body mass index

^a^Used at least three out of the five strategies to engage communities

^b^Intervention included changes to the physical environment

^c^Statistically significant results reported

^d^Results not statistically significant

^e^No statistical test conducted

### Quality appraisal of studies

Study quality was assessed using the Effective Public Health Policy Project (EPHPP) Quality Assessment Tool. The EPHPP Quality Assessment Tool provides criteria to evaluate studies on the basis of selection bias, study design, confounders, blinding, data collection methods, withdraws and dropouts, intervention integrity, and analysis. Each criterion is scored numerically according to the guidelines as strong (score = 1), moderate (score = 2), or weak (score = 3). Subsequently, the entire article is rated as strong (no weak ratings), moderate (one weak rating), or weak (two or more weak ratings).

## Results

### Program objectives

Program objectives varied. Four focused on reducing BMI, one focused on increasing physical activity, five focused on improving diet or food behaviors, six focused on improving knowledge/awareness and/or self-efficacy, five focused on improving other health outcomes (e.g., Hgb A1c, blood pressure, cholesterol), and 18 studies had multiple objectives.

### Program activities

All programs had a nutrition education component. Seven also focused on changes to the physical environment (Table 1). For example, an obesity prevention trial in American Indian children incorporated changes to foods served in the school setting to reduce calories [9]. In another study in Native American high school youth, an existing room in the high school was remodeled into a fitness center [10]. Twenty-nine programs included a physical activity component in addition to the dietary component.

### Target populations

Sixteen programs focused on groups suffering from a chronic condition, such as overweight/obesity or diabetes. Twenty-four programs focused exclusively on adults, seven programs focused exclusively on children, and seven programs included a wide age range. Nineteen studies focused exclusively on American Indian populations. Seven studies included AI and/or AN in addition to one or more ethnic groups. None focused on Alaska Native populations exclusively. Information on the target population is reported in Table 1.

### Geographic region

With regards to geographic region, two programs were conducted in the Northeast, six in the Midwest, three in the South, 22 in the Western region, and six in more than one region of the US.

### Formative research to inform design

Twenty-five programs reported conducting formative research to inform the design of the intervention, although the nature and duration of the formative research varied. While in some cases investigators relied on literature review or review of existing data to inform design, in most cases, the community was directly involved in this step. Formative activities included interviewing or conducting focus groups with members of the target population for guidance regarding the content, format, and method of delivery of the intervention, pilot testing of the intervention in the community, and review of the curriculum by the community. For example, the *Strong in Body and Spirit* program in Native Americans involved focus group sessions in the community that allowed members to express their desire for inclusion of traditional foods and values [11]. The types of formative research used to inform design of the intervention in each study are displayed in Table 2.

**Table 2:** Strategies used in nutrition interventions conducted in Indigenous populations in the US: Formative research to inform design

	Story et al. [9]^a^	Brown et al. [47]^a^	Gittelsohn et al. [45]^a^	Caballero et al. [55]^a^	Hill et al. [17]^b^	Bachar et al. [37]^b^	Mendenhall et al. [42]^b^	Buller et al. [43]^b^	Gittelsohn et al. [53]^b^	Weaver et al. [12]^b^	Gellert et al. [48]^b^	Sinclair et al. [54]^b^	Murphy et al. [13]^b^	Mau et al. [14]^b^	Richards et al. [66]^b^	Jiang et al. [67]^b^	Karanja et al. [72]^b^	Witmer et al. [74]^b^	DeWeese et al. [75]^b^	Driscoll et al. [76]^b^	Rinderknecht et al. [16]^b^	Thompson et al. [78]^b^	Gilliland et al. [11]^b^	Jernigan et al. [69]^c^	Ritenbaugh et al. [10]
Strategy																									
Formative research to inform design																									
Focus groups/ interviews with community members	✓	✓	✓	✓		✓	✓	✓	✓	✓		✓	✓	✓	✓		✓	✓	✓	✓	✓		✓	✓	✓
Pilot testing of intervention in community											✓			✓		✓						✓			
Review of curriculum by community					✓																				

^a^Randomized controlled trial

^b^Pre-post study

^c^Post test

### Formative research to inform evaluation

Five programs identified included formative research to inform evaluation of the intervention. These activities included collaborative work of the research team with the community to identify outcome measures, as well as examination of evaluation instruments by members of the target population to ensure relevance. For example, for the *Healthy Living in Two Worlds* project conducted in urban Native youth, a draft of the instrument to be used to assess knowledge, attitudes and behaviors related to tobacco use, dietary practices and physical activities was evaluated by several Haudenosaunee youth to insure that the questions were clear and meaningful to this particular population [12]. The types of formative research used to inform evaluation of the intervention in each study are displayed in Table 3.

**Table 3:** Strategies used in nutrition interventions conducted in Indigenous populations in the US: Formative research to inform evaluation

	Caballero et al. [55]^a^	Mendenhall et al. [42]^b^	Weaver et al. [12]^b^	Richards et al. [66]^b^	Jernigan et al. [69]^c^
Strategy					
Formative research to inform evaluation					
Research team worked with community to identify outcome measures		✓			✓
Evaluation instruments reviewed by members of target population	✓		✓	✓	

^a^Randomized controlled trial

^b^Pre-post study

^c^Post test

### Partnership

Twenty-eight programs involved a partnership between researchers and the community. Such partnerships involved collaboration with the target population on data collection, collaboration with local organizations or establishments, development of a community action plan with the target population, delivery of the intervention by members of the target population, holding regular meetings with the target population, and collaboration with the tribal review board or ethics committee. For example, in a type 2 diabetes prevention intervention in Native American communities, program staff networked with local agencies to share information and resources to inform the intervention [13]. The types of partnerships formed in each study are displayed in Table 4.

**Table 4:** Strategies used in nutrition interventions conducted in Indigenous populations in the US: Partnership

	Story et al. [9]^a^	Brown et al. [47]^a^	Kattelmann et al. [50]^a^	Gittelsohn et al. [45]^a^	Caballero et al. [55]^a^	Hill et al. [17]^b^	Bachar et al. [37]^b^	Beckham et al. [39]^b^	Mendenhall et al. [42]^b^	Bradley et al. [44]^b^	Gittelsohn et al. [53]^b^	Carrel et al. [46]^b^	Benyshek et al. [49]^b^	Burden et al. [51]	Mau et al. [14]	Sinclair et al. [54]^b^	Murphy et al. [13]^b^	Mau et al. [14]^b^	Richards et al. [66]^b^	Jiang et al. [67]^b^	Moore et al. [68]^b^	Howard et al. [70]^b^	Gilliland et al. [11]^b^	Ritenbaugh et al. [10]	DeWeese et al. [75]^b^	Rinderknecht et al. [16]^b^	Smith et al. [77]^b^	Jernigan et al. [69]^c^
Strategy																												
Partnership																												
Collaboration with target population on data collection	✓																											
Collaboration with local organizations or establishments			✓		✓	✓				✓	✓	✓		✓		✓	✓			✓	✓		✓	✓	✓		✓	✓
Development of community action plan with target population							✓																					
Delivery of intervention by members of target population				✓				✓					✓		✓			✓										
Regular meetings with target population		✓							✓																	✓		
Collaboration with tribal review board/ethnics committee																			✓			✓						

^a^Randomized controlled trial

^b^Pre-post study

^c^Post test

### Capacity building

Twenty-one programs included some aspect of capacity building among project staff and community members in their programs. Examples of capacity building included training community members to perform health-promoting practices, use of “train the trainer” sessions, joint development of a community action plan, formation of local working groups, and promotion of career development of project staff. For example, several studies used community peer educators to deliver lessons that formed part of the intervention [14, 15]. The types of capacity building that were part of each study are displayed in Table 5.

**Table 5:** Strategies used in nutrition interventions conducted in Indigenous populations in the US: Capacity building

	Story et al. [9]^a^	Brown et al. [47]^a^	Kattelmann et al. [50]^a^	Gittelsohn et al. [53]^a^	Caballero et al. [55]^a^	Hill et al. [17]^b^	Bachar et al. [37]^b^	Beckham et al. [39]^b^	Mendenhall et al. [42]^b^	Buller et al. [43]^b^	Weaver et al. [12]^b^	Benyshek et al. [49]^b^	Sinclair et al. [54]^b^	Murphy et al. [13]^b^	Mau et al. [14]^b^	Richards et al. [66]^b^	Karanja et al. ^b^	Harvey-Berino et al. [15]^b^	Smith et al. [77]^b^	Thompson et al. [78]^b^	Jernigan et al. [69]^c^
Strategy																					
Capacity building																					
Community members trained to perform health-promoting practices	✓	✓	✓	✓	✓			✓	✓	✓	✓	✓	✓		✓		✓	✓	✓	✓	✓
Train-the-trainer sessions incorporated						✓															
Joint development of community action plan							✓														
Local working group formed														✓		✓					
Promotion of career development of project staff					✓																

^a^Randomized controlled trial

^b^Pre-post study

^c^Post test

### Involvement of local food system

Sixteen programs incorporated some aspect of the local food system into the intervention. This included involving local food service or local food retailers in the intervention, as well as incorporating local foods into the educational materials and/or activities programs. For example, in an after-school intervention of urban Native American youth, discussion of the importance and roles of traditional foods of the group under study formed part of lessons provided [16]. The types of involvement of the local food system in each study are displayed in Table 6.

**Table 6:** Strategies used in nutrition interventions conducted in Indigenous populations in the US: Involvement of local food system

	Story et al. [9]^a^	Kattelmann et al. [50]^a^	Gittelsohn et al. [45]^a^	Beckham et al. [39]^b^	Mendenhall et al. [42]^b^	Buller et al. [43]^b^	Bradley et al. [44]^b^	Gittelsohn et al. [53]^b^	Gellert et al. [48]^b^	Jiang et al. [67]^b^	Gilliland et al. [11]^b^	Leslie et al. [73]^b^	DeWeese et al. [75]^b^	Driscoll et al. [76]^b^	Rinderknecht et al. [16]^b^	Ritenbaugh et al. [10]^b^
Strategy																
Involvement of Local Food System																
School food service involved in intervention	✓															✓
Local foods incorporated in educational materials and/or activities		✓		✓	✓	✓	✓	✓	✓	✓	✓		✓	✓	✓	
Local food retailers involved in intervention			✓				✓	✓				✓				

^a^Randomized controlled trial

^b^Pre-post study

### Outcomes

Thirty–one programs of the 39 programs (79%) identified reported significant changes in knowledge, behavior or health (Table 1). In terms of the degree of community involvement in the studies reporting significant changes in outcomes, all except one program included at least one of the five strategies examined in this review regarding community engagement, and 19 used at least three out of the five strategies (Table 1). For example, the Cancer 101 program employed formative research to inform the design of the program in working with tribal health directors to review the curriculum, demonstrated partnership with organizations that had established relationships with tribal communities and involved capacity building in the form of train the trainer sessions [17]. In further examining the studies reporting significant results, five were RCTs, 24 were pre-post studies, one had a multiple cross-sectional design, and one was a retrospective study.

### Study quality

Twelve studies were rated as strong, 13 studies were rated as moderate, and 14 studies were rated as weak according to the EPHPP Quality Assessment Tool.

## Discussion

Results of the current literature review yielded research studies conducted with a variety of objectives in diverse Indigenous groups throughout the US. Overall, interventions were successful in producing changes in knowledge, behavior or health. The degree to which communities were involved in the design, implementation, and evaluation of the intervention varied from not at all to involvement at all stages. Of the programs reporting significant changes in outcome measures, more than half used at least three strategies to engage communities. The extent of use of strategies to promote community engagement in programs reporting significant outcomes is notable.

While formative research to inform the design of the interventions was performed in most studies, formative research to inform the evaluation was not performed to a great degree, with only six programs identified including this step. In some of these cases, researchers worked with the community to determine what outcomes should be measured, a process that enhances the program’s alignment with traditional values. In particular, following an indigenous evaluation framework, as described by LeFrance and colleagues, ensures that programs pay sufficient attention to the cultural context in which behaviors arise, thereby increasing the potential success of interventions [18]. In other cases, members of the target population provided input on the content of evaluation tools and participated in pilot testing. Previous research has revealed the importance of this step and has outlined procedures that may be used to determine face validity of instruments [19], as well as other procedures that should be conducted to evaluate the psychometric properties of evaluation tools [20]. It is essential that evaluation tools are designed in collaboration with the target audience to ensure relevance [20]. Given that very few studies identified in this review involved formative research to inform evaluation, it is possible that not all relevant outcomes were reported. While results were often presented in terms of changes in weight and diet, there may be other outcomes such as cultural connectedness that may be important for the communities in question but were not measured.

Fewer than half of the programs identified described involvement of the local food system. In further seeking to promote health in Indigenous communities, incorporation of local foods is an important consideration. Working in concert with local food retailers or other establishments may allow for the inclusion of traditional foods in the intervention. In communities in which people identify themselves with their culture and natural environment, use of traditional food systems to improve health builds community support and engagement for holistic health and well-being [21]. Factors contributing to obesity and diabetes in Indigenous people are complex, and are rooted in dietary Westernization that leads to significant changes in food sources and nutrient intake [22]. In Indigenous communities, participating in the traditional food system is linked to higher diet quality, lower levels of chronic disease risk factors, lower levels of stress and overall well-being. The traditional diet of Yup’ik People in Southwestern Alaska, for example, is rich in vitamin D, vitamin A, vitamin E, iron, and n-3 fatty acids through inclusion of foods such as fatty fish, fish roe, seal oil and wild game [22]. Similarly, the traditional diet of Native Hawaiians has been found to be high in fiber, polyunsaturated fatty acids, and low in fat and cholesterol [23]. Over the past century, Indigenous food systems have been compromised as a result of social, economic, political and environmental pressures [24]. The consequences of the transition away from nutrient-rich traditional food in Indigenous communities include shifts in nutrients intake, obesity and associated diet-related chronic conditions [25–27]. In a study of Pima Indians in Arizona, for example, intake of complex carbohydrate, dietary fiber, insoluble fiber, vegetable proteins and the proportion of total calories from complex carbohydrate and vegetable proteins were significantly higher in the Indian than in the Anglo diet [28]. Intervention activities must work in concert with the local cultural and social settings, local personnel and local sources of food [21].

The majority of programs were carried out in the Western region. Of note, of the studies conducted in this region, relatively few were focused on populations in Alaska and Hawai’i. As Indigenous groups in these states face health disparities, future studies may further focus on these geographic areas. Among Native Hawaiians, for example, the prevalence of chronic diseases constituting the leading causes of death in the U.S.—heart disease, cancer, and diabetes—is notably high [29]. Rates of diet-related chronic conditions such as cardiovascular disease are also high in Alaska Natives [30]. Other researchers have pointed to the need for individual-level interventions for behaviors related to obesity in groups such as Native Hawaiians [29].

Also of note, most programs focused exclusively on adults. As particular nutrition-related burdens fall on youth, children continue to be an important target group in further addressing issues related to diet in Indigenous groups. According to data from the National Health and Nutrition Examination Survey (NHANES), 17% of children and adolescents in the U.S. were obese in 2011–2014 [31]. Previous studies indicate that the diet quality of children and adolescents in the U.S. falls short of the recommendations, and that diet quality may be improved by increased intake of foods such as vegetables, whole grains and seafood [32]. Good nutrition is essential for child growth and development, as well as for maintenance of a healthy weight [33].

Ten of the interventions identified were randomized controlled trials, and half of these reported significant results. Most studies used a pre-test, post-test design. Without a control group, such studies do not provide the same level of evidence as the RCT and do not allow conclusions of the same caliber to be drawn [34]. However, given that research in Indigenous populations may be performed in small, geographically dispersed samples, it may not always be feasible to conduct a RCT. Studies with a pre-test, post-test design may provide evidence to inform additional studies in the population of interest. Of note, there were a number of studies rate as “weak” with regards to study quality. These ratings reflected issues such as failure to report withdrawals and drop-outs, as well as lack of assessment of validity and reliability of data collection tools. Failure to report withdrawals and dropouts leads to difficulty in determining the degree to which participants engaged in the intervention and the effects of the program, as evaluation of all participants enrolled in the program may not have been possible. In other cases, it was not possible to ascertain whether the outcome assessors were aware of the intervention or exposure status of participants or if the study participants were aware of the research question. These issues may have compromised study results to varying degrees. If, for example, outcome assessors were aware of the intervention or exposure status of participants, this may have led them to alter their behavior toward participants or perform measurements differently in the two groups. In cases in which less than 100% of participants received the allocated intervention, it is possible that there may have been systematic differences between those who completed the intervention and those who did not, compromising the results of the study.

With regards to program activities, relatively few programs involved changes to the physical environment. However, of those that did, all found significant results, and five out of the seven studies also involved used of at least three strategies to engage communities. Other researchers have noted the need to design interventions at multiple levels, including individual, social environmental, physical environmental, and macrosystem [35]. While all of the programs identified in this review had a nutrition education component, a change in health outcomes may not result without concomitant environmental changes. In planning future interventions, this is an important consideration.

### Limitations

While not all studies made use of the strategies identified in this review to engage communities, it should be noted that not all studies focused exclusively on Indigenous populations. In some cases, the target population was comprised of both Indigenous people and members of other groups. In these instances, it may not have been possible or reasonable to use some of the strategies identified, such as incorporating traditional foods into the intervention. This should be taken into account in examining results. Further, only one database was used to identify articles to include in the review, and gray literature was not examined. Thus, it is possible that some relevant articles may not have been located.

## Conclusions

Interventions performed in Indigenous groups in the US were generally successful in producing changes in knowledge, behavior or health. Interventions mostly targeted adults in the Western region and used a pre-test, post-test design. Of the nutrition interventions reporting significant changes in outcome measures, more than half involved notable engagement of communities. The degree of use of strategies to promote community engagement in programs reporting significant outcomes is notable. However, formative research to inform the evaluation was not performed to a great degree, and fewer than half of the programs identified described involvement of the local food system. In planning interventions in Indigenous groups, researchers may consider the range of ways in which the community may be involved in the design, execution and evaluation of the intervention based on the studies reviewed here. Such involvement is likely to improve the success of these initiatives. There is a particular need for studies focused on Indigenous youth in diverse regions of the US to further address diet-related chronic conditions.

## Abbreviations

AI: American Indian
AN: Alaska Natives
BMI: Body mass index
CBPR: Community-based participatory research
CHW: Community health worker
NA: Native American
NH: Native Hawaiian
NHOPI: Native Hawaiian and Other Pacific Islander
RCT: Randomized controlled trial
